# Polymeric Microparticles of Calcium Pectinate Containing Urea for Slow Release in Ruminant Diet

**DOI:** 10.3390/polym13213776

**Published:** 2021-10-31

**Authors:** Myrla Melo, André da Silva, Edson Silva Filho, Ronaldo Oliveira, Jarbas Silva Junior, Juliana Paula Oliveira, Antônio Vaz, José Moura, José Pereira Filho, Leilson Bezerra

**Affiliations:** 1 Department of Animal Science, Federal University of Campina Grande, Avenida Universitária, s/n-Jatobá, Patos 58708-110, Brazil; myrla_kristy@hotmail.com (M.M.); juli.foo@gmail.com (J.P.O.); antonio.fernando@professor.ufcg.edu.br (A.V.); jose.fabio@ufcg.edu.br (J.M.); jmpfpiaui@gmail.com (J.P.F.); 2 Chemistry Department, Federal University of Piaui, Ininga, s/n, Teresina 64049-550, Brazil; edsonfilho@ufpi.edu.br; 3 Department of Animal Science, Federal University of Bahia, Av. Adhemar de Barros, 500, Ondina, Salvador City 40170-110, Brazil; ronaldooliveira@ufba.br (R.O.); miguelreges@gmail.com (J.S.J.)

**Keywords:** biochemistry, slow release, pH and rumen temperature, protozoa

## Abstract

In ruminant feeding, mechanisms for controlling the rate of ammonia release in the rumen are important for increasing the efficiency of transforming dietary nitrogen into microbial protein. Three microencapsulated formulations, with increased urea concentrations of 10 (MPec1), 20 (MPec2) and 30% (MPec3) from the *w*/*w*, based on the mass of citrus pectin solution, employ the external ionic gelation/extrusion technique. The properties of microencapsulated urea were examined as a completely randomized design with 5 treatments each with 10 replicates for evaluation, and the ratios of dietary to free urea were compared using 5 fistulated male Santa Ines sheep in a Latin 5 × 5 square design. The degradation kinetics showed that the rate of controlled release from the microencapsulated systems was significantly reduced compared with that of free urea (*p* < 0.05). The population density of ruminal protozoa increased when sheep received the microencapsulated urea (*p* < 0.05). The disappearance of dry matter and crude protein reached a degradation plateau during the first minutes for the MPec1 and MPec2 systems and was slower for MPec3. The MPec1 and MPec2 systems presented higher (*p* < 0.05) blood serum concentrations of albumin, urea nitrogen (BUN), creatinine and total cholesterol and did not affect (*p* > 0.05) the other blood metabolites. The MPec2 systems are recommended because they consist of microspheres with more (*p* < 0.05) controlled core release, delaying the peak of urea released in the rumen and BUN without affecting (*p* < 0.05) ruminal pH and temperature. Microencapsulation with calcium pectinate provided better utilization of urea, reducing the risk of ruminant intoxication.

## 1. Introduction

The use of urea ((NH_2_)_2_CO) as a source of nonprotein nitrogen (NPN) has been applied on a large scale in ruminant diets, mainly to reduce production costs. (NH_2_)_2_CO is hydrolyzed by ureolytic bacteria in the rumen into ammonia nitrogen (NH_3_-N) and incorporated by ruminal microorganisms for microbial protein production, which is very important to ruminants [1]. The NH_3_-N peak in the rumen when urea is supplied usually occurs 1 to 2 h after feeding, while for true protein sources, this peak occurs approximately 3 to 5 h after feeding [1,2]. However, there is concern about its use at high levels in animal diets due to the low utilization of NH_3_-N by ruminal microorganisms and, especially, the possibility of the intoxication of the ruminant herd [3].

The low palatability of urea and its intoxication capacity when converted to ammonia in the rumen by ruminal microorganisms limits its inclusion in concentrate mixtures to 2% or 40 g/100 kg body weight, and the supply to the animals must be conducted in covered troughs to avoid volatilization [4,5].

The speed of ammonia release in the rumen is the determining factor in the transformation of dietary nitrogen into microbial protein [6,7], and the adaptation of the ruminal environment of animals to urea is essential. Thus, the urea microencapsulation technique can gradually release this ingredient in the rumen environment, reducing the risk of animal poisoning and improving the synchronism of nutrients in the rumen without compromising productive performance [8]. Several materials are being tested as encapsulating systems with very promising results [6,9,10].

One of these materials is citrus pectin, a heteropolysaccharide contained in the middle and primary lamellae and cell walls of land plants [11,12], which has attracted significant interest in the food industry [13]. Chemically, pectins are biopolymers composed of homogalacturonan, xylogalacturonan and rhamnogalacturonans, with the latter being divided into two types: rhamnogalacturonans І and rhamnogalacturonans ІІ [11,14]. The chemical composition of citrus pectin allows its ability to form gels due to the differences in the size of the polygalacturonic acid chain and the degree of esterification of its carboxylic groups, which can be used in the production of several materials as it is a natural product and easy to acquire [12].

Shtriker et al. [15] observed that intake from diets containing soluble fibers from citrus pectin resulted in gut microbiota comprising a healthier flora and led to positive effects on weight, glycemic control and liver β oxidation via adenosine monophosphate-activated protein kinase in mice. Ben-Ghedalia et al. [16] investigated a pectin-rich diet in sheep and its effect on rumen parameters and nitrogen (N) metabolism. It was observed that the dried citrus pectin pulp can provide favorable conditions for cellulolysis in the rumen with a positive effect on the supply of N to the intestine. Thus, if used as an encapsulating agent, citrus pectin might allow the protection of urea, promoting its slower release, improving its use and reducing environmental contamination by N excretion from the urea cycle.

The rapid degradation of urea in the rumen by microbial urease, with risk of intoxication due to the formation of ammonia, as well as the need for the adaptation of animals fed diets containing urea, have motivated studies to develop products that allow the gradual release of urea in the ruminal environment to increase the production of microbial protein and reduce intoxication cases [17,18]. Therefore, the technology of the microencapsulation of feed has become an interesting option because it allows a greater synchronism of the degradation rate of this compound in the rumen [6,9,10], keeping the NH_3_-N concentration constant over a long period and, consequently, allowing a greater use of the feed by the animal [19]. Thus, the aim was to test the hypothesis that calcium pectinate can be used as a microencapsulation agent for efficient and slow release of urea and to increase the addition of urea in the sheep diet without harming the microflora and the ruminal environment, as well as reducing the risk of intoxication to the sheep.

## 2. Materials and Methods

### 2.1. Obtaining and Characterization of Microencapsulated Systems

The microencapsulated formulations experiment was examined as a completely randomized design with 5 treatments each (free urea, microencapsulated urea into calcium pectinate at different levels (MPec1, MPec2 and MPec3) and calcium pectinate encapsulating matrix free as a control) with 10 replicates.

Three microencapsulated formulations from calcium pectinate containing urea (U) were developed using citrus pectin as an encapsulating agent with a fixed concentration of 5% (*w*/*v*), defined from a previous test, and increased urea concentrations of 10 (MPec1), 20 (MPec2) and 30% (MPec3) from the *w*/*w*, based on the weight of citrus pectin solution, employing the external ionic gelation/extrusion technique.

To prepare the respective solutions, the corresponding masses of urea and pectin were duly weighed for each formulation and dissolved in distilled water. Then, for each system, the urea solution was slowly added to the pectin solution and stirred with a glass rod until completely homogenized. Each solution of the core/encapsulant mixture was subsequently extruded with the aid of a plastic syringe in a previously prepared 3% (*w*/*v*) calcium chloride crosslinking bath to form calcium pectinate microparticles. The extrusion was carried out from a fixed height of 10 cm, and the microspheres remained in contact with the crosslinking solution for 30 min under constant magnetic stirring centrifuged at 400× *g*. Finally, the microspheres were separated with the aid of a sieve, washed with distilled water, transferred to a plastic tray and dried in an oven at 45 °C for 24 h.

Subsequently, micrographs of calcium pectinate microparticles with and without urea were obtained by optical microscopy, in a Medilux^®^ microscope (Barneveld, The Netherlands) and by stereomicroscopy. For scanning under an optical microscope, the samples were fixed on a cover slip with adjusted lighting and 40× magnification.

The microencapsulation yield was based on the masses of urea and pectin solution before and after ionic extrusion/gelation, calculated using the following equation:

*MY* = (*MF*/*MI*) × 100 (1)

where *MY* = microencapsulation yield; *MF* = final mass of the microencapsulated product after extrusion/crosslinking; and *MI* = initial mass of urea and pectin solution.

The microencapsulation efficiency evaluated the retention capacity of the calcium pectinate matrix and was determined based on the urea content inserted and the content retained after the process. The microencapsulation efficiency was calculated using the following equation:

*ME* = (*Uactual*/*Utheoretical*) × 100 (2)

where *ME* = microencapsulation efficiency; *Uactual*: actual retained urea content; *Utheoretical*: Urea content inserted.

Urea was quantified according to the AOAC Kjeldahl method [20]. The data obtained were analyzed to quantify the total nitrogen using the following equation:

%*N* = *V* × *M* × *F* × 0.014 × 100/m (3)

where *M* = molarity of hydrochloric acid, 0.02 N; *F* = hydrochloric acid correction factor = 1.00; 0.014, milliequivalent weight of nitrogen (g); *V* = volume of hydrochloric acid used in the titration, in mL; m = sample weight (g).

Thermogravimetry (TG) and differential scanning calorimetry (DSC) curves for urea, calcium pectinate and microencapsulated systems were obtained simultaneously in a thermal analyzer (SDT Q600, V20.9 Build 2, Columbus, OH, USA), under an inert atmosphere, flow of 100 mL/min, heating rate of 10 °C/min, from 30 to 600 °C, using a platinum crucible containing approximately 8.0 mg of sample. T_onset_ was considered to evaluate the thermal stability of the materials studied from the TG curves. The temperature peaks were considered to extract the events from DSC curves.

### 2.2. Ethical Considerations, Animals, Diets and General Procedures

The experimental trial was developed in strict accordance with the recommendations contained in the Guide of the National Council for the Control of Experiences in Animals, Brazil, and the protocol was approved by Permit Number 116/2018 [21].

Five rumen-fistulated sheep (initial average body weight (BW) 30.4 ± 6 kg) and age of 28 ± 2 months housed in individual pens (1.0 × 1.5 m) fitted with feeders and waterers were assigned in a 5 × 5 Latin square design, with five consecutive 16-day periods divided into 14-day adaptation and 2-day (48 h) sampling periods. All animals were previously treated for internal and external parasites with ivermectin (Ivomec gold, Merial^®^, Salvador, Bahia, Brazil) and vaccinated against clostridiosis (Sintoxan, Merial^®^, Sao Paulo, Brazil). 

The formulated diets were prepared according to National Research Council (NRC) [22] guidelines for sheep maintenance and growth with a weight gain of 40 g/d and an average weight of 30 kg. The sheep received water ad libitum and were fed twice daily (08:00 and 16:00 h) with total mixed ration (TMR). The feed refusals were collected and weighed daily, and the amount of feed offered was adjusted to allow a 100 g/kg refusal. The chemical composition and proportion of ingredients and experimental diets are presented in Table 1. The diet provided presented ≅ 10.5% crude protein (CP) and ≅35% non-fibrous carboydrates (FC), meeting synchronism of energy and protein [22] and an amount of fiber (≅50) meeting the minimum amount for rumination of the animals [23].

Samples of the ingredients and refusals were pre-dried in a forced-air ventilation oven at 55 °C for 72 h. Then, samples of the ingredients and refusals were ground in a Wiley knife mill with a sieve size of 1 mm. The samples were stored in plastic jars with lids, labeled and subjected to analyses (triplicate) to determine the dry matter (DM; method 967.03), ash (method 942.05), crude protein (CP; method 981.10) and ether extract (EE; method 920.29) contents [20]. Analyses for the determination of neutral detergent fiber (NDF) and acid detergent fiber (ADF) were performed according to Van Soest et al. [23], with changes proposed by Senger et al. [24] to include the use of an autoclave. The autoclave temperature was maintained at 110 °C for a period of 40 min. The nonfiber carbohydrates (NFCs) were determined by the following equation calculated by Hall [25] (2000):

*NFC* = 100 − [(*CP* − *CP from urea* + *urea*) + *NDF* + *EE* + *Ash*. (4)

### 2.3. Degradation Kinetics and Ruminal and Blood Serum Parameters

The in situ technique was used for ruminal degradation from the nonwoven fabric bags ((TNT—100 g/m^2^ (polypropylene)) with dimensions of 4.5 × 4.5 cm, sealed by a sealing machine. Each bag contained approximately 1.0 g of sample (microencapsulated urea into calcium pectinate at different levels (MPec1, MPec2 and MPec3) and free urea and calcium pectinate encapsulating matrix free as a control). Each bag and sample were oven-dried at 55 °C for 24 h and weighed to determine the dry matter content.

During the incubation period, the samples in the bags corresponded to three treatments with the MPec produced, free urea and calcium pectinate, placed in duplicate for each sheep (5) at the respective time intervals (0.25, 0.5, 1, 3, 6, 12, 24 and 48 h), as well as a blank, with two bags per time interval (8) for each animal, totaling 16 bags (identified with graphite). The samples were placed at the final time (48 h), and gradually, the remaining samples were placed, so that the contents spent their time in the rumen and at the end they were all removed at once. The bags for time (0 h) zero (used to determine the readily soluble fraction) were introduced into water for 20 s and then removed, receiving the same treatment as the others. In adaptation period, four bags were inserted during 48 h as control (blank) and were used to determine the ruminal content that adheres to the bags for subsequent correction of the dry matter Afterward, they were washed in running water, placed in an oven with forced ventilation at 55 °C for 72 h and then weighed on an analytical balance. The degradation profiles were calculated by the nonlinear least-squares approach using the exponential model described by Ørskov and McDonald [26].

The in situ ruminal degradation kinetics of dry matter (DM) and nitrogen (N) were calculated using the exponential model of Ørskov and McDonald [26]:

*PD* = *a* + *b* [1 − e ^(−*c* × *t*)^] (5)

where *PD* is the potential degradation at time t; *a* is the readily water-soluble fraction; *b* is the water-insoluble but potentially degradable fraction; *c* is the fraction degradation rate *b*; and *t* is the incubation time in minutes. After processing and incubation of the samples, the nitrogen profile was analyzed according to the AOAC Kjeldahl method [20].

The effective DM and N degradability (*ED*) in the rumen was calculated using the following equation:

*ED* = *a* + [(*b* × *c*)/*c* + *k*)] (6)

where *a* is the soluble fraction, *b* is the potentially degradable insoluble fraction, *c* is the degradation rate constant and *k* is the passage rate of the digesta from the rumen. The ED was calculated using a single rumen-reticulum particle passage rate of 8%/h. The undegradable fraction (*U*) was calculated from *a* and *b* fractions:

*U* = 100 − (*a* + *b*) (7)

For the ruminal parameters, all sampling of the ruminal content was performed during the collection of bags. The degradability test was performed at each time of incubation of the microencapsulated samples: 0, 0.25, 0.5, 1, 3, 6, 12, 24 and 48 h. The collection of ruminal fluid occurred manually at different locations in the rumen through the fistula, obtaining a sample of approximately 200 mL. After each collection, the content went through the filtration process, which was separated into containers (20 mL) with a specific amount for each parameter evaluated. The pH and temperature were immediately measured with the aid of a pH meter and a portable digital thermometer that was previously calibrated.

Each sample collector contained 2 mL of filtered inoculum and 4 mL of M.S.F solution (35% formaldehyde, methyl green and sodium chloride). To carry out protozoan counting, the samples were initially homogenized with the aid of a magnetic stirrer, after which the reading was carried out in a Neubauer chamber in which 10 µL was pipetted into each counting area of the chamber and a cover slip was placed on top to improve the visualization of protozoa. The reading was carried out under an optical microscope (Lumen) at 40× in the C field. In the center of these chambers, there were several perpendicular lines with markings in quadrants, so there were 4 readings in each quadrant. The final count results were calculated by equation:

*N* × 3 × 10,000 = *NP*/1 mL (8)

where *N*: mean of the readings of quadrant C (uppercase) in mL; 3: inoculum dilution; 10,000: constant; *NP*: protozoan population count.

### 2.4. Blood Metabolites

The blood collection procedure of the five animals was performed by jugular venipuncture from each animal in tubes using a vacuum system (Becton, Dickson and Co., São Paulo, SP, Brazil) at the same incubation times of the degradability test. Blood collection occurred simultaneously with the ruminal incubation times of the microencapsulated systems. One #16 catheter (Medical supply^®^, Sao Paulo, Brazil) was inserted in each animal to facilitate collection and meet animal welfare requirements. Samples were temporarily kept at room temperature until clot retraction and then centrifuged (Centrifuge 90–1 model, Coleman^®^, São Paulo, Brazil) at 2500× *g* for five min to generate blood serum. Finally, the serum was stored at −20 °C in Eppendorf Tubes^®^ (Sigma-Aldrich, São Paulo, Brazil) until analysis. The serum metabolites were measured using commercial kit tests to measure total protein, albumin, blood urea nitrogen (BUN), creatinine, aspartate aminotransferase (AST), cholesterol and triglycerides from the means obtained in an automated Cobas C111 biochemical apparatus (Roche, Ludwigsburg, Germany) in enzymatic or colorimetric kinetic assays. The electrolytic analysis was performed in an automated analyzer (Max Ion-Medmax, Shenzhen, China) by the direct ion selectivity method for calcium, chlorine, sodium and potassium.

### 2.5. Statistical Analysis

The microencapsulated systems were examined as a completely randomized design with 5 treatments (MPec1, MPec2 and MPec3, free urea and calcium pectinate) and 10 replicates. For evaluation of ruminal parameters and blood parameters, a 5 × 5 Latin square design (5 treatments and 5 periods) was used, and the results were analyzed and measurements were repeated over time (0, 0.25, 0.5, 1, 3, 6, 12, 24 and 48 h relative to the incubation time) using the MIXED procedure of SAS [27] following a model including experimental treatment, incubation time, animal and interactions according to following Equation:

*Yijk* = *m* + *Ti* + *aj* + *Pk* + *eijk*, (9)

where *Yijk* is the dependent variable measured in animal *j*, which was subjected to the *i* treatment in period *k*; *μ* is the general mean; *Ti* is the fixed effect of treatment *I*; *aj* is the random effect of animal *j*; *Pk* is the random effect of period *k*; and *eijk* is the unobserved random error assuming normal distribution. All means were compared using the Tukey test, with a critical level of 5% probability being adopted for type I error. 

## 3. Results

### 3.1. Characterization of Microencapsulated Systems

**Figure 1:**
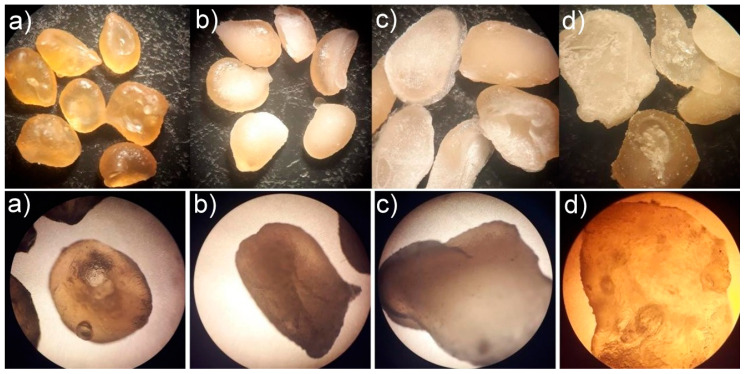
Micrographs obtained by stereomicroscope (Top) and optical microscope (Bottom) of (**a**) calcium pectinate and microencapsulated systems MPec1 (**b**), MPec2 (**c**) and MPec3 (**d**).

The microencapsulated systems and calcium pectinate (empty pectin microparticles) were evaluated for their microstructure under stereoscopic and optical microscopy. The micrographs (Figure 1) demonstrated that compared to calcium pectinate microparticles, all systems were whitish in color due to the presence of urea. The image of the three systems showed no porosity or cracks, although MPec1 and MPec2 have a more regular particle shape. The MPec3 microencapsulated system showed a rough surface, which suggests the incidence of surface urea.

The addition of urea from the microencapsulated systems to calcium pectinate increased (*p* ≤ 0.05) the microencapsulated yield to 92.2, 93.3 and 97.1% for MPec1, MPec2 and MPec3, respectively (Table 2). There was a reduction (*p* ≤ 0.05) in the encapsulation efficiency with the increasing addition of urea, with values of 262, 218 and 264% and actual urea retention of 25.2, 28.4 and 31.1% for MPec1, MPec2 and MPec3, respectively.

**Figure 2:**
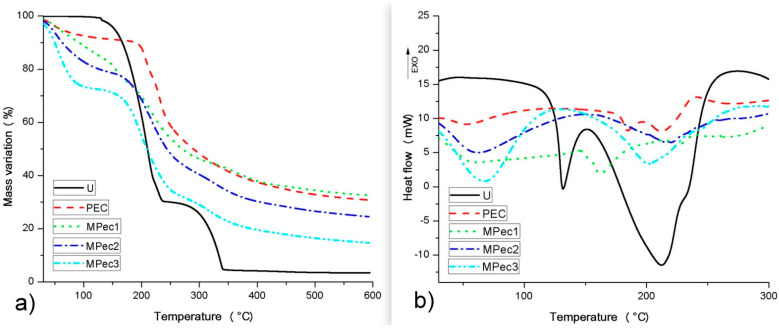
(**a**) Thermogravimetric (TG) and (**b**) differential scanning calorimetry (DSC) curves of the bioactive films obtained from the incorporation of Cashew Nut Shell Liquid into a matrix of sodium alginate.

The TG curves (Figure 2a) demonstrated in general that urea, calcium pectinate and the microencapsulated systems presented two stages of mass loss, the first one due to moisture loss in the case of pectin and microparticles. The DSC curves (Figure 2b) show an endothermic event below 100 °C for calcium pectinate and the microencapsulated systems, corresponding to moisture loss, as also seen in the TG curves. Urea showed endothermic events at 130 and 212 °C, corresponding to its melting and thermal degradation, respectively. Pectin showed two endothermic events at 184 and 213 °C and one exothermic event at 240 °C, corresponding to its thermal degradation. A higher melting temperature (162 °C) was observed for the urea in MPec1 compared to free urea (130 °C), while the urea in MPec2 and MPec3 did not show melting events, suggesting simultaneous melting and thermal degradation.

### 3.2. Degradation Kinetics and Ruminal and Blood Serum Parameters

There was a greater disappearance (*p* ≤ 0.05) of the initial soluble fraction (a) of DM in the MPec3 treatment compared to other microencapsulation systems and calcium pectinate and a smaller disappearance of the crude protein fraction (a) in the MPec3 treatment (Table 3).

Regarding the potentially degradable insoluble fraction (b), the behavior was the opposite; the greatest disappearance (*p* ≤ 0.05) of DM occurred in pectinate calcium encapsulating matrix free, which differed only from the microencapsulated system with 30% urea (MPec3), while the fraction (b) of crude protein disappearance was higher (*p* ≤ 0.05) in MPec3 compared to the other microencapsulated systems, which formed similar (MPec1 and MPec2) and had a greater disappearance of fractions (b) in relation to pectinate. Regarding the insoluble fraction (U), there was a greater disappearance of DM (*p* ≤ 0.05) in pectinate and MPec1, which was superior to the MPec2 and MPec3 treatments. In contrast, the disappearance of fraction (U) of the major crude protein in MPec3 differed from pectinate and the other two formulations.

The DM effective degradation (ED) did not differ between treatments. However, the highest ED of CP was observed for calcium pectinate encapsulating matrix free, followed by the respective additions of microencapsulated urea (MPec1, MPec2 and MPec3).

**Figure 3:**
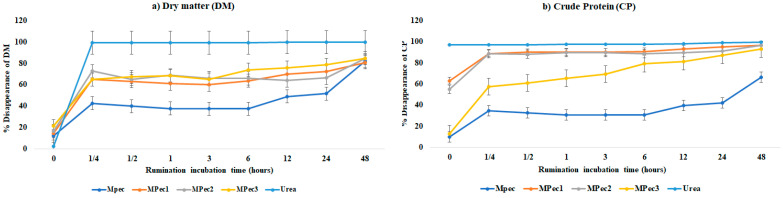
Mean disappearance of (**a**) dry matter (DM) and (**b**) crude protein (CP) in sheep from the urea and encapsulating matrix (MPec) free and microencapsulated urea in calcium pectinate matrix (MPec1, MPec2 and MPec3) incubated in sheep rumen at different times (Significant when *p* < 0.05).

Lower DM and CP disappearances (potential degradation—PD) were observed in calcium pectinate encapsulating matrix free, and there was no difference between the PD of the microencapsulated systems (Figure 3).

Disappearance reached the degradation plateau at 0.25 h and then remained constant until 48 h of incubation, when all treatments reached a disappearance peak above 85%. There was a greater DM disappearance in free urea, as expected, and within 0.25 h, >99% of the urea had already disappeared confirming of the rapid disappearance after the first minutes of ruminal incubation.

**Figure 4:**
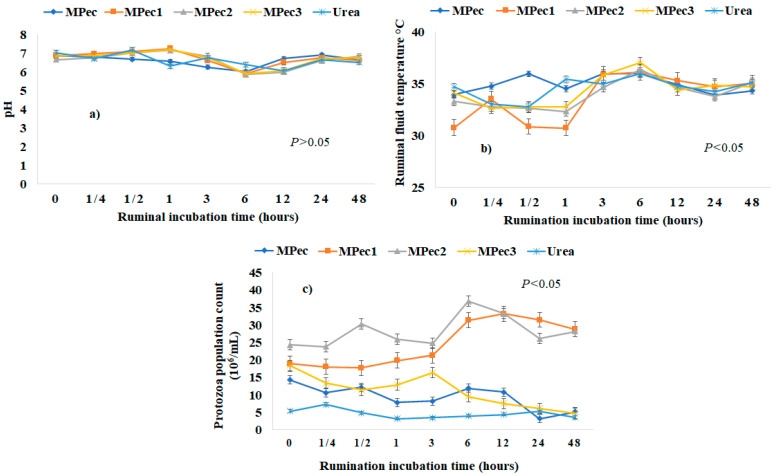
Mean variation of ruminal (**a**) pH; (**b**) ruminal temperature; and (**c**) total count of protozoa from incubation of urea and encapsulating matrix (MPec) free and microencapsulated urea in calcium pectinate matrix (MPec1, MPec2 and MPec3) in sheep rumen at different times.

There was no interaction (*p* > 0.05) between the treatments and the ruminal incubation for rumen pH (Figure 4a). The use of microspheres containing urea in their cores did not affect ruminal pH, with mean values varying between 7.02 and 7.26 between time periods from 0.5 and 1 h.

There was an effect of the treatments on the mean temperature values (*p* ≤ 0.05) of the ruminal fluid (Figure 4b). At the incubation times of 0, 0.5 and 1 h, MPec1 promoted significant changes (*p* ≤ 0.05) at the other times evaluated compared to the other treatments, while MPec3 stood out at a higher temperature (37 °C) compared to the other microencapsulated systems (*p* ≤ 0.05), urea and encapsulating matrix free, with a significant effect at 0.25, 0.5 and 1 h. Calcium pectinate, free urea and MPec2 did not differ in temperature as a function of collection time. Likewise, it was evident that free urea did not differ significantly from the three microencapsulated systems or from calcium pectinate.

Regardless of the collection time, the inclusion of MPec1 followed by MPec2 presented the higher (*p* ≤ 0.05) counts of protozoa at time periods between 0 and 48 h, and the higher counts (peak) occurred at 6 h. These two systems present practically the same protozoa count at 48 h. There was no difference (*p* > 0.05) in the protozoan counts between the calcium pectinate encapsulating matrix free, MPec3 and urea treatments (Figure 4c). For the inclusion of MPec3 in the rumen of sheep, the number of protozoa in the rumen environment was reduced compared to the other two systems.

The MPec1 and MPec2 microencapsulated systems presented higher blood serum concentrations of albumin, nitrogen (BUN), creatinine and total cholesterol (Table 4) in sheep compared to MPec3 and to free urea, which did not differ from others treatments (*p* ≤ 0.05).

**Figure 5:**
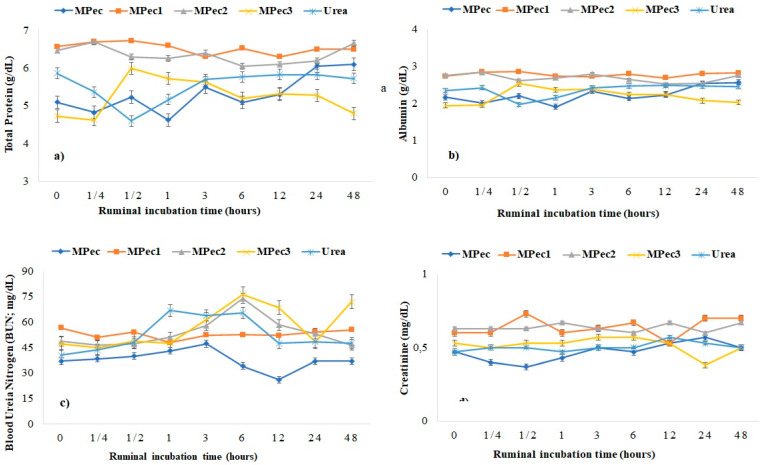
Mean and standard deviation (SD) of serum concentrations of (**a**) total protein; (**b**) albumin; (**c**) BUN; and (**d**) creatinine in blood sheep from incubation of urea and encapsulating matrix (MPec) free and microencapsulated urea in calcium pectinate matrix (MPec1, MPec2 and MPec3) at different times.

The total protein presented a greater serum concentration in the MPec1 and MPec2 microencapsulated systems (Table 4), with total protein values of 6.73 and 6.67 mg/dL, respectively, at 0.25 h (Figure 5a). There was a decrease (*p* ≤ 0.05) in albumin values (Figure 5b) for the inclusion of MPec3 treatment at time periods 0 and 0.25 h (1.95 and 1.96 g/dL, respectively), as well as in the use of free urea at the time period of 0.5 h and for calcium pectinate encapsulating matrix free at the time period of 1 h, presenting the lowest blood concentration observed (1.91 g/dL).

The peak of BUN occurred at six hours after incubation for the MPec2 and MPec3 treatments (*p* ≤ 0.05), while for the other treatments, this peak occurred at three hours after incubation (Figure 5c). The lowest value (*p* ≤ 0.05) was observed for serum creatinine concentration in sheep (Figure 5d) for the use of free calcium pectinate encapsulating matrix free at 0.25 h, with 0.40 mg/dL, and the highest concentration (0.73 mg/dL) was at 0.5 h in the MPec1 system.

The concentrations of triglycerides and calcium, chlorine, potassium and sodium electrolytes were not affected (*p* > 0.05) by the microencapsulated systems (MPec1, MPec2 and MPec3) or by encapsulating matrix free and urea. The microencapsulated system MPec3 (43.6 U/L) had a lower concentration (*p* ≤ 0.05) of AST enzymes than the system with free urea (65.9 U/L), but there was no difference between the other treatments. There was no significant effect (*p* > 0.05) for the enzyme AST in relation to the incubation time.

## 4. Discussion

All microencapsulated systems showed a high microencapsulation yield, indicating that external ionic gelation is an adequate technique for urea microencapsulation, and citrus pectin was shown to be a viable encapsulation matrix. Noh et al. [28], in their study of microencapsulating multiple hydrophobic and hydrophilic active agents, described the potential use of pectin in microcapsule formulations as protection of active agents by gelation by electrostatic crosslinking.

Regarding the values of microencapsulation efficiency over 100%, the actual urea increase is related to the microencapsulation technique used, since in the microsphere drying process, the water present is evaporated and the core content is concentrated. It was observed that the microencapsulation efficiency decreased as the urea content increased, indicating an advantage for the lower levels inserted. This is because each encapsulating material has a retention limit, as well as the influence of the aqueous medium for preparing the microparticles, in which there may already be an early release of urea given its high solubility in water. Nevertheless, all three systems showed good results. When evaluating the microencapsulation efficiency of urea as a nucleus, Medeiros et al. [6] and Carvalho Neto et al. [10] obtained values above 98%.

It was observed from the micrographs that the higher the urea content inserted, the more irregular, thinner and larger the particle became, assuming a flattened shape. Morphological evaluations are important factors to study the protection of the encapsulated content, its uniformity in size and its release conditions. This is because the effectiveness of the use of microspheres directly depends on the properties of the microencapsulating agent, which should not allow the release of the nucleus before the desired time [6,7].

T_onset_ is the most commonly used parameter to estimate a material’s thermal stability and is considered the point where thermal degradation begins. According to the data extracted from the TG curves (Figure 2a), free urea presented T_onset_ at 164 °C, a value approximately that (170.5 °C) found by Carvalho et al. [9]. The urea in microencapsulated systems showed the beginning of thermal degradation at a higher temperature when compared to free urea, considering the T_onset_, whose values were 180, 181 and 169 °C for MPec1, MPec2 and MPec3, respectively. The DSC curves confirmed a better thermal stability for urea after microencapsulation. This showed the effectiveness of urea protection by citrus pectin in the form of calcium pectinate encapsulating matrix free, especially to MPec1 and MPec2, which presented more effective protections against thermal degradation, probably due to the influence of the particle microstructure (more regular and thicker), according to the visual analysis itself and as already indicated in the micrographs. Urea in all microencapsulated systems had better thermal stability compared to free urea, with emphasis on MPec1 and MPec2, whose core protections can be reproduced in the rumen, enabling gradual release and, therefore, better use and less risk of intoxication.

Urea microencapsulation from the citrus pectin promoted a gradual disappearance of the DM (Figure 3) after the first 25 min, regardless of the system, lower than urea and greater than pectinate. From 0.25 h, there was a disappearance of the stability of material for both DMs. This behavior indicates that it was released more slowly in the rumen, because, in the first 25 min, the free urea was almost totally released, whereas in the microencapsulated system, the release above 80% only occurred at 48 h.

Despite the fact that protozoa make up a large portion of the rumen biomass, their role in ruminal fermentation and their contribution to the metabolism and nutrition of the host is still an area of substantial controversy [29]. The higher concentration of ammonia promoted by free urea and MPec3 is probably the most consistent factor to explain protozoal elimination and seems to be due to decreased bacterial protein breakdown and feed protein degradability in the absence of protozoan rumen [29,30]. Moreover, it has been demonstrated that although bacterial predation by rumen protozoa is dependent on the protozoal size, a lower protozoa population is beneficial due to an increase in bacteria population and can result in lower methane emission, because one of the many important symbiotic associations formed in the rumen is that of the relationship between methanogenic archaea and ciliated protozoa [31].

Regarding free urea, in the early time periods (0.25 and 0.5 h), almost all the incubated content disappeared, with values of approximately 99.9 and 99.6%, respectively, which differs from microencapsulated systems in which up to 48 h showed degradability of 80.8, 84.7 and 84.7% for MPec1, MPec2 and MPec3, respectively, indicating that all treatments showed a gradual release. Furthermore, when we analyzed BUN, it was observed that MPec2 and MPec3 were delayed, reaching the highest blood concentrations only after 6 h of incubation, whereas MPec1 and free urea peaked 3 h after incubation. These results confirm that citrus pectin was a suitable wall material to protect urea through the acquisition of calcium pectinate microparticles, since the protection promoted a delay in ruminal release, as well as in serum levels of BUN. According to Patra and Aschenbach [5], the forage takes about 4 to 6 h to start its degradation, while urea, after 4 h of ruminal incubation, practically disappears. This statement corroborates our findings, in which the urea disappeared practically in the first 15 min, probably due to being inserted in the bag without feed mixture. However, when more pectinate was added in the formulation (30%), a slower release occurred.

In general, the MPec1 and MPec2 systems showed a more gradual degradation rate after 3 h, suggesting a more effective protection for urea. This may have occurred due to the higher core content of the MPec3 system, since it increases the possibility of urea being closer to the particle surface and therefore being more easily released/degraded. DM disappearance increases with the concentration of urea used in the system because the increase in urea content increases the amount of NPN that is highly soluble in water.

The lower values of the indigestible fraction (U) of crude protein can be explained because the microencapsulated systems had a higher value of the soluble fraction. The MPec1 and MPec2 systems remained similar, while MPec3 presented a higher value, expressing its low degradation potential compared to the other studied systems. Regarding the degradation rate, the MPec2 system presented the lowest values, with 0.35 and 3.57% for DM and crude protein, respectively. Faster passage rates favor greater efficiency in the growth of ruminal microorganisms. The MPec3 presented a lower soluble CP and ED of CP than MPec1 and MPec2, implying that MPec3 can release urea slowly but has more bypass urea to the lower gut. While MPec1 has more soluble CP and a higher ED of CP, this implies that MPec1 releases urea faster (a = 63.1%) than MPec3 (a = 12.7%), and the majority of urea in MPec1 is degraded in the rumen (ED = 80%).

The use of microspheres containing urea in their core only interfered with the ruminal pH in a gradual way, with mean values varying between 7.02 and 7.26 between times from 0.5 and 1 h, with no significant changes between the evaluated treatments. It is important to emphasize that regardless of the system, the lowest pH values were observed at 3 h and 6 h. Van Soest [3] states that for an environment favorable to bacterial proliferation, it is necessary that the ruminal pH ranges between 6.0 and 7.0 and that a pH ranging between 5.5 and 6.0 significantly reduces the activity of the ruminal microbiota in the rumen. The MPec1 and MPec2 systems presented the best ruminal pH ranges.

From the characterizations and visual analyses, MPec3 showed a lower capacity to protect the nucleus, which favors the rapid release of nitrogen, consequently implicating in microbial growth [32]. It was observed that the number of protozoa per milliliter significantly varied between the studied treatments and incubation times, noting that the use of more citrus pectin in microencapsulated systems favored the increase in the population density of protozoa, because it probably enabled a more favorable ruminal environment, since ammonia in larger quantities slows the growth of microorganisms [4,33]. These results indicate that the gradual release of urea promotes better efficiency in the use of nitrogen and the maintenance of a stable environment, since the use of free urea exhibited inferior and differentiated behavior for the conditions of ruminal adaptation. Ammonia is produced from dietary protein, or urea is used by the ruminal microorganisms for their growth, which is subsequently available to the host as a microbial protein [5].

The MPec1 and MPec2 systems had the highest serum concentrations of total protein, albumin and creatinine, probably due to the greater amount of encapsulating matter, thus allowing for a faster rate of ammonia release compared MPec3 but in a smaller amount. This provided a more favorable ruminal environment for the growth of microorganisms and allowed a greater count of the protozoan population [5]. Urea is synthesized in the liver in amounts proportional to the concentration of ammonia produced in the rumen, and its concentration is directly related to the protein levels in the feed and the energy–protein ratio in the diet [2,34]. Calomeni et al. [2], quantifying the effects of feeding polymer-coated slow-release urea on blood parameters, noted similar values for albumin analysis (2.37 and 2.34 mg/dL) and higher creatinine values (1.26 and 1.27 mg/dL) for slow-release urea and conventional urea, respectively.

The addition, the MPec3 system increased the concentration of BUN, probably due to the higher concentration of urea in the microencapsulated system, and, consequently, a greater production of ruminal ammonia and the greater difficulty microorganisms have in using ammonia [4,5,33]. The consequence was a reduction in the total microorganism count. The serum urea concentration in the MPec3 treatment was at the maximum threshold (58 mg/dL) of the stipulated physiological standards (23 to 58 mg/dL) for the sheep breed [2,34,35]. Ziguer et al. [36], for instance, found average values of 62.45 and 63.82 mg/dL. Other research using the microencapsulation of urea from other materials has shown the range of serum urea in sheep to be greater compared to the literature [6,9,37]. Perhaps this is due to the longer continuing release of urea into the rumen [7,9,37]. However, it is still too early to state from only published articles that this is the range for the use of slow-release urea. It is noteworthy that no animal showed signs of toxicity, but the excess of free ammonia in the rumen is undesirable, as it can lead to energy losses by the animal to release this ammonia through the urine [9,34]. According to Kozloski [38], blood urea has a positive correlation with the concentration of ammonia in the rumen and with the use of amino acids (alanine, glutamine and glycine) in the liver. Calomeni et al. [2] observed mean values of 45.5 mg/dL with the use of microencapsulated slow-release urea in the sheep diet.

## 5. Conclusions

The calcium pectinate microparticles containing urea studied in sheep diet for slow release improved effective and potential degradability and protozoa population count, without affecting blood metabolite concentrations. The MPec2 formulation presented microspheres with a more controlled urea release. Thus, the use of citrus pectin as a urea wall material to obtain calcium pectinate microencapsulated systems is a promising alternative, as it can provide a better use of urea, reduce the risk of animal poisoning, as it did not change the liver metabolic enzymes, and favor the safe administration of larger amounts of this ingredient to ruminants.

## Figures and Tables

**Table 1:** Chemical composition and proportion of ingredients and experimental diets containing levels microencapsulated urea into calcium pectinate matrix of formulations (MPec1, MPec2 and MPec3).

Item	Ingredients							
Chemical Composition (g/kg DM)	Tifton-85 Hay	Soybean Meal	Ground Corn	MPec1	MPec2	MPec3	MPec ^3^	Urea
Dry matter (g/kg as fed)	872	907	889	990	990	990	990	-
Ash	60.3	70.1	15.1	-	-	-	-	-
Crude protein	54.1	489	93.9	740	796	875	77.3	2800
Ether extract	10.8	54.9	30.1	24.3	24.3	24.3	24.3	-
Neutral detergent fiber	728	198	158	219	219	219	219	-
Acid detergent fiber	371	95.2	43.0	-	-	-	-	-
Nonfibrous carbohydrate	138	487	751	59.5	59.5	59.5	59.5	-
	Experimental diets							
Ingredient proportion (g/kg DM)	Urea	MPec1		MPec2	MPec3		MPec	
Tifton-85 hay	600	600		600	600		600	
Soybean meal	60.0	80.0		80.0	80.0		90.0	
Ground corn	325	305		305	305		295	
Encapsulation material ^1^	5.00	5.00		5.00	5.00		5.00	
Mineral mixture ^2^	10.0	10.0		10.0	10.0		10.0	
Chemical composition (g/kg DM)								
Dry matter (g/kg as fed)	881	882		882	882		882	
Ash	55.2	56.3		56.3	56.3		5.68	
Crude protein	106	104		104	105		105	
Ether extract	19.6	2.02		2.02	2.02		2.04	
Neutral detergent fiber	500	501		501	501		502	
Acid detergent fiber	243	244		244	244		244	
Nonfibrous carbohydrate	356	354		354	354		351	

^1^ Urea free, pectinate or microencapsulated urea at different levels. ^2^ Presenting guaranteed the following levels of active elements: 120 g calcium, 87 g phosphorus, 147 g sodium, 18 g sulfur, 590 mg copper, 40 mg cobalt, 20 mg chromium, 1.8 g iron, 80 mg iodine, 1.3 g manganese, 15 mg selenium, 3.8 g zinc, 300 mg molybdenum and a maximum of 870 mg fluoride. Solubility of phosphorus citric acid: 2 to 95%. ^3^ Encapsulating matrix based on citrus pectin produced on the basis of ionic gelation/extrusion technique.

**Table 2:** Mean and standard deviation (±SD) of the total nitrogen, crude protein, theoretical and actual urea contents and microencapsulation efficiency of microencapsulated urea into calcium pectinate matrix of formulations (MPec1, MPec2 and MPec3).

Variables (%)	Microencapsulated Urea			*p*-Value ^1^
	MPec1	Mpec2	Mpec3	
Microencapsulation yield	92.2 ^a^ ± 0.02	93.3 ^b^ ± 0.03	97.1 ^c^ ± 0.04	<0.01
Microencapsulation efficiency	262 ^a^ ± 1.17	218 ^b^ ± 1.12	264 ^a^ ± 1.16	<0.01
Nitrogen (N) total	11.2 ^a^ ± 0.08	12.8 ^b^ ± 0.09	14.0 ^c^ ± 0.10	<0.01
Crude protein	70.1 ^c^ ± 0.88	80.2 ^b^ ± 1.06	87.5 ^a^ ± 0.79	<0.01
Theoretical urea content	10.0 ^c^ ± 0.0	20.0 ^b^ ± 0.0	30.0 ^a^ ± 0.0	<0.01
Actual urea content	25.2 ^b^ ± 0.48	28.4 ^a^ ± 0.32	31.1 ^a^ ± 0.30	<0.01

^1^ Means followed by the different letters differ (a; b and c) differ from Tukey’s test when *p* ≤ 0.05.

**Table 3:** Rumen degradation profiles in sheep fed with microencapsulated urea intro calcium pectinate matrix of formulations (MPec1, MPec2 and MPec3) and encapsulating matrix (Mpec).

Variables	Microencapsulated Urea				*p*-Value
	MPec1	MPec2	MPec3	MPec	
Dry matter (DM)					
a ^1^ (%)	14.5 ^b^ ± 3.42	16.5 ^b^ ± 1.49	21.1 ^c^ ± 6.58	11.9 ^a^ ± 3.61	<0.01
b ^2^ (%)	66.2 ^ab^ ± 12.00	68.2 ^ab^ ± 1.55	63.5 ^b^ ± 12.26	70.1 ^a^ ± 6.14	<0.01
U ^3^ (%)	19.2 ^a^ ± 5.88	15.3 ^b^ ± 3.04	15.4 ^b^ ± 5.90	18.0 ^a^ ± 8.02	<0.01
c ^4^ (%h^−1^)	4.48 ^a^ ± 2.37	0.35 ^c^ ± 0.25	0.66 ^c^ ± 0.25	2.67 ^b^ ± 0.89	<0.01
ED ^5^ (%)	36.4 ± 11.25	19.2 ± 3.74	25.9 ± 5.86	29.1 ± 8.02	0.34
Crude Protein (CP)					
a ^1^ (%)	63.1 ^b^ ± 18.02	56.3 ^b^ ± 7.92	12.7 ^c^ ± 4.10	97.3 ^a^ ± 0.05	<0.01
b ^2^ (%)	33.6 ^b^ ± 10.95	40.6 ^b^ ± 8.92	79.5 ^a^ ± 10.58	2.10 ^c^ ± 0.33	<0.01
U ^3^ (%)	3.33 ^ab^ ± 1.04	3.15 ^ab^ ± 1.12	7.81 ^a^ ± 6.11	0.56 ^b^ ± 0.27	<0.01
c ^4^ (%h^−1^)	7.17 ^b^ ± 3.93	3.57 ^c^ ± 0.69	9.08 ^a^ ± 1.80	9.30 ^a^ ± 4.87	<0.01
ED ^5^ (%)	80.1 ^b^ ± 3.66	68.5 ^c^ ± 6.63	54.7 ^d^ ± 0.74	98.2 ^a^ ± 0.58	<0.01

Means followed by the different letters differ (a; b and c) from Tukey’s test when *p* ≤ 0.05; ^1^ soluble/rapidly degradable fraction; ^2^ slowly degradable fraction; ^3^ undegradable fraction; ^4^ degradation rate (c) of fraction “b” expressed in %/h; ^5^ effective degradation considering a pass rate of 8%/h). MPec is encapsulating matrix free based on citrus pectin produced on the basis of ionic gelation/extrusion technique.

**Table 4:** Mean and standard deviation (SD) of blood serum metabolites in sheep fed with microencapsulated urea into calcium pectinate matrix of formulations (MPec10, MPec2 and MPec3) and urea and calcium pectinate encapsulating matrix (MPec) free.

Metabolites	Microencapsulated Urea			Free		*p*-Value
	MPec1	MPec2	MPec3	Urea	MPec	
Albumin (g/dL)	2.78 ^a^ ± 0.11	2.69 ^a^ ± 0.20	2.21 ^b^ ± 0.35	2.36 ^b^ ± 0.24	2.23 ^b^ ± 0.32	0.03
Total protein (g/dL)	6.53 ^a^ ± 0.27	6.35 ^a^ ± 0.45	5.23 ^b^ ± 0.78	5.54 ^b^ ± 0.61	5.32 ^b^ ± 0.81	<0.01
BUN ^3^ (mg/dL)	52.7 ^b^ ± 6.49	53.7 ^b^ ± 8.17	58.0 ^a^ ± 9.13	52.4 ^b^ ± 11.26	37.8 ^c^ ± 6.28	<0.01
Creatinine (mg/dL)	0.64 ^a^ ± 0.09	0.64 ^a^ ± 0.14	0.53 ^b^ ± 0.14	0.50 ^b^ ± 0.10	0.47 ^b^ ± 0.10	0.028
Cholesterol (mg/dL)	29.0 ^a^ ± 5.87	29.4 ^a^ ± 8.81	22.8 ^b^ ± 6.09	24.5 ^b^ ± 5.11	22.6 ^b^ ± 3.62	<0.01
Triglycerides (mg/dL)	14.9 ± 6.40	16.1 ± 3.78	14.2 ± 4.63	13.4 ± 4.85	15.1 ± 5.43	0.64
Calcium (mmol/L)	2.68 ± 0.39	2.72 ± 0.46	2.93 ± 0.22	2.76 ± 0.31	2.64 ± 0.35	0.53
Chlorine (mmol/L)	110 ± 6.19	110 ± 5.12	112 ± 3.25	115 ± 17.26	109 ± 16.92	0.55
Potassium (mmol/L)	5.58 ± 2.57	8.64 ± 6.41	10.3 ± 3.15	4.97 ± 2.45	4.67 ± 0.99	0.48
Sodium (mmol/L)	141 ± 9.72	136 ± 9.38	132 ± 3.18	144 ± 21.68	136 ± 21.50	0.23
AST ^4^ (U/L)	59.2 ^ab^ ± 13.31	53.7 ^ab^ ± 10.40	41.6 ^b^ ± 10.66	65.9 ^a^ ± 11.52	57.1 ^ab^ ± 10.69	<0.01

Means followed by the different letters differ (a; b and c) from Tukey’s test when *p* ≤ 0.05; ^3^ Blood urea nitrogen; ^4^ Aspartate aminotransferase.

## Data Availability

The data that support the findings of this study are available from the corresponding author upon reasonable request.
